# Ionophore-Based Potentiometric Sensors for the Flow-Injection Determination of Promethazine Hydrochloride in Pharmaceutical Formulations and Human Urine

**DOI:** 10.3390/s110101028

**Published:** 2011-01-18

**Authors:** Ahmed Khudhair Hassan, Bahruddin Saad, Sulaiman Ab Ghani, Rohana Adnan, Afidah Abdul Rahim, Norariza Ahmad, Marina Mokhtar, Suham Tawfiq Ameen, Suad Mustafa Al-Araji

**Affiliations:** 1 Department of Chemistry, College of Science, Baghdad University, Baghdad, Iraq; E-Mails: ahmedkhh71@yahoo.com (A.K.H.); souadmustafa24@yahoo.com (S.M.A.-A.); 2 School of Chemical Sciences, Universiti Sains Malaysia, 11800 Penang, Malaysia; E-Mails: sag@usm.my (S.A.G.); r_adnan@usm.my (R.A.); afidah@usm.my (A.A.R.); nriza@hotmail.com (N.A.); marimokh79@gmail.com (M.M.); 3 Department of Chemistry, College of Science, Tikrit University, Tikrit, Iraq; E-Mail: drsuhaam_t@yahoo.com (S.T.A.)

**Keywords:** flow injection analysis, ionophore, potentiometric sensor, promethazine

## Abstract

Plasticised poly(vinyl chloride)-based membranes containing the ionophores (α-, β- and γ-cyclodextrins (CD), dibenzo-18-crown-6 (DB18C6) and dibenzo-30-crown-10 (DB30C10) were evaluated for their potentiometric response towards promethazine (PM) in a flow injection analysis (FIA) set-up. Good responses were obtained when β- and γ-CDs, and DB30C10 were used. The performance characteristics were further improved when tetrakis(4-chlorophenyl) borate (KTPB) was added to the membrane. The sensor based on β-CD, bis(2-ethylhexyl) adipate (BEHA) and KTPB exhibited the best performance among the eighteen sensor compositions that were tested. The response was linear from 1 × 10^−5^ to 1 × 10^−2^ M, slope was 61.3 mV decade^−1^, the pH independent region ranged from 4.5 to 7.0, a limit of detection of 5.3 × 10^−6^ M was possible and a lifetime of more than a month was observed when used in the FIA system. Other plasticisers such as dioctyl phenylphosphonate and tributyl phosphate do not show significant improvements in the quality of the sensors. The promising sensors were further tested for the effects of foreign ions (Li^+^, Na^+^, K^+^, Mg^2+^, Ca^2+^, Co^2+^, Cu^2+^, Cr^3+^, Fe^3+^, glucose, fructose). FIA conditions (e.g., effects of flow rate, injection volume, pH of the carrier stream) were also studied when the best sensor was used (based on β-CD). The sensor was applied to the determination of PM in four pharmaceutical preparations and human urine that were spiked with different levels of PM. Good agreement between the sensor and the manufacturer’s claimed values (for pharmaceutical preparations) was obtained, while mean recoveries of 98.6% were obtained for spiked urine samples. The molecular recognition features of the sensors as revealed by molecular modelling were rationalised by the nature of the interactions and complexation energies between the host and guest molecules.

## 1. Introduction

**Figure 1.**
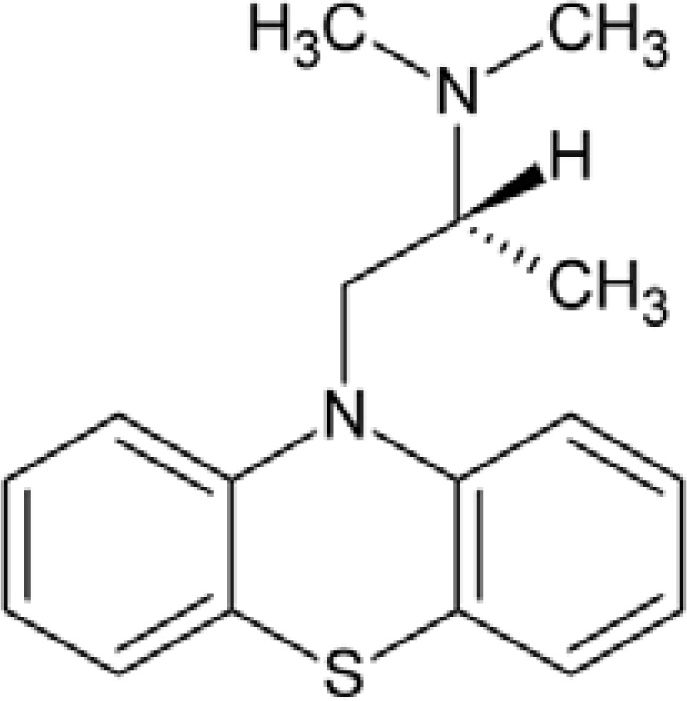
Structure of promethazine.

Promethazine hydrochloride (PM) [(*RS*)-*N,N*-dimethyl-1-(10*H*-phenothiazine-10-yl)propan-2-amine hydrochloride] is a phenothiazine derivative (Figure 1). It is a first generation H1 receptor antagonist, antihistamine and antiemetic medication and can also have strong sedative effects [1–3]. The function of PM is to block histamine H1 receptors without blocking the secretion of histamine.

Several methods including high-performance liquid chromatography (HPLC) [1–4] capillary electrophoresis [5,6] spectrophotometry [7] chemiluminescence [8] and voltammetry [9] have been used for the determination of PM. Recently, the flow injection analysis (FIA) technique combined with different detection systems such as spectrophotometry [10,11] fluorimetry [12] and potentiometry [13] have been used for the assay of PM in pharmaceutical formulations. However, most of these methods are complicated, time consuming and also require expensive instrumentation. The spectrophotometric methods in particular suffer from interference problems if no effective pretreatment is in place. The oxidised form of PM is unstable which requires a rapid and fully automated reagent handling technique [10–12]. Thus, there is a need for simpler, low-cost, sensitive and rapid alternative methods for the determination of PM in real samples.

Recent developments, especially in the late 1990s, have clearly shown the great promise of the potentiometric sensor (PS) for use with real samples. The dramatic lowering of detection limits to 10^−8^–10^−11^ M, polyion sensing [14,15] sensors for neutral analytes, *etc.* are some of these exciting developments. PS’ have found diverse applications including environmental [16] and drug analysis [17].

Several reports on the development and application of PM sensors for the determination of PM in pharmaceutical preparations [18–20] can be found in the literature. These are all based on ion-pairing agents that are plagued by limited selectivity, thus their applications are restricted to samples with simple matrices such as pharmaceutical preparations. For PM sensors to be applicable in more challenging matrices (e.g., real body fluids), a more selective molecular recognition component is clearly required. To this end interesting PS’, capitalising on the selective host-guest inclusion complexes using ionophores such as calixarenes [21], crown ethers and cyclodextrins (CDs) [22–25] for the sensing of drugs have been reported.

This study aims in developing a simple, rapid and selective PM sensor that can be used for the determination of PM in real samples. The strategy was to explore the PM sensing capabilities of ionophores such as CDs (α-, β- and γ-CD) and crown ethers (DB18C6 and DB30C10), and exploiting the best system as a flow-through detector in a FIA set-up. Molecular modelling was also used to investigate the nature of the host-guest interaction(s).

## 2. Materials and Methods

### 2.1. Reagents and Chemicals

All chemicals were of analytical grade and used as received. High-molecular-weight poly(vinyl chloride) (PVC), potassium tetrakis(4-chlorophenyl)-borate (KTPB), dibenzo-18-crown-6 (DB18C6), and tetrahydrofuran (THF) were obtained from Fluka AG (Switzerland). PM hydrochloride, dioctylphenylphosphonate (DOPP), bis(2-ethylhexyl) adipate (BEHA), dibenzo-30-crown-10 (DB30C10), α-, β-, γ-CD and tri-*n*-butyl phosphate (TBP) were purchased from Sigma-Aldrich (USA). PM syrups and tablets were purchased from local pharmacies. Doubly deionised water was used to prepare all solutions. A stock solution of 0.1 M PM was prepared by dissolving an appropriate amount of PM in 50 mL water. Standard solutions (1 × 10^−2^ to 1 × 10^−6^ M) PM were prepared fresh by diluting the appropriate amount of PM in water. Each stock solution was protected from light by wrapping the flasks with aluminium foil and stored in a refrigerator when not in use. Stock solutions (0.1 M) of various salts and sugars were also prepared. Other solutions were prepared by the subsequent dilutions of the stock solutions.

### 2.2. Apparatus

**Figure 2.**
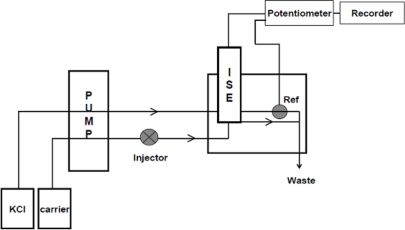
Schematic diagram of the FIA manifold.

The FIA system (Figure 2) consisted of a multi-channel peristaltic pump (Gilson Miniplus 3) and an injection valve (Rheodyne Type 500 Teflon rotary selection valve), with an exchangeable sample loop. The rest of the FIA tubings were of 0.80 mm i.d. A sampling volume of 150 μL and carrier flow rate of 2.83 mL min^−1^ were used throughout. The flow-through cell was of wall-jet design with a built-in Ag/AgCl reference electrode (Model FIP-3, Chemflow Devices, Australia) [26]. The sample was injected into a carrier stream of acetate buffer (0.1 M, pH 6.0). Potassium chloride (0.1 M) was used as reference solution. Potentiometric measurements were performed at 25 ± 2 °C using an Orion model 701A digital ionanalyser that was connected to the flow cell. A glass electrode, connected to an Orion expandable ionanalyser (model EA 940) was used to measure the pH of aqueous solutions.

### 2.3. Preparation of the Promethazine Sensor

Potentiometric PM selective sensors was prepared by dissolving PVC (176.0 mg), plasticising solvent (356.4 mg), ionophore (11.0 mg) and KTPB where appropriate (6.6 mg) in THF (5 mL). Four drops of the mixture were deposited on the electrode body [26] and the system was allowed to evaporate for 3 h. The membrane was then conditioned in 1 × 10^−2^ M PM for 1 h prior to use. PM standard solutions covering the range 1×10^−6^ to 1×10^−1^ M was injected into the FIA unit. Each solution was measured in triplicate. The average potentials at maximum heights were plotted against log [PM].

### 2.4. Selectivity

Selectivity coefficients, $K_{A,B}^{\text{pot}}$, of the fabricated sensors towards several cations and sugars were measured using the separate solution method [27] and the following equation was applied:

(1) $$\text{log}\;K_{A,B}^{\text{pot}}=\frac{E_{B}-E_{A}}{S}+\left[1-\frac{Z_{A}}{Z_{B}}\right]\;\text{log}\;a_{A}$$

where *E*_A_ and *E*_B_ are the potential readings of PM and interferring ions, respectively; a_A_ is the activity or concentration of PM. *Z*_A_ and *Z*_B_ are the charge of PM and interferring ions, respectively and *S* is the slope of the calibration graph.

### 2.5. Determination of PM in Pharmaceutical Preparations and Human Urine

The United States Pharmacopoeia method [28] was used for the determination of PM in certain samples as comparison to the proposed method.

#### 2.5.1. Tablets

A homogenised powder was prepared from ten accurately weighed tablets. An appropriate amount of this powder was dissolved in water in a 50 mL Erlenmeyer flask. The dissolution of the drug was assisted by means of a magnetic stirrer and by an ultrasonic bath. The mixture was then filtered and made up to the mark with water in a 100 mL volumetric flask.

#### 2.5.2. Syrup

An appropriate amount of syrup containing 1 and 3 mg mL^−1^ of PM (as claimed by the manufacturers) was diluted with doubly deionised water in a 50 mL Erlenmeyer flask.

#### 2.5.3. Human Urine

Appropriate volumes of standard PM solution were spiked into 10 mL Erlenmeyer flasks, and was topped-up to the mark with urine of healthy student volunteers.

### 2.6. Computational Methods

**Figure 3.**
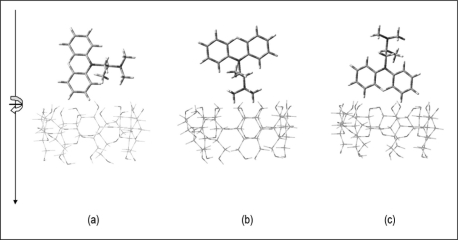
The three different orientations considered for the insertion of promethazine into the cavity of the host molecules with the N atom from the alkyl group of promethazine moving in **(a)** vertically, **(b)** facing up, and **(c)** down horizontally.

The starting geometries of PM and the host structures (α-CD, β-CD, γ-CD, DB18C6 and DB30C10) were built based on the structures that were generated from the crystallographic parameters provided by the Cambridge Structural Database (CSD) [29–33] and were separately optimised using the semi empirical method, PM3 using Gaussian03 software package [34]. The starting geometries of the inclusion complexes were constructed using HyperChem (Version 7.0, Hypercube, Gainesville, FL, USA). The previously optimised structures of PM and host molecules were allowed to approach each other along the symmetric axis (the X-axis) passing through the center of the host cavity. For example, in the case of β-CD, the coordinate system used to define the process of complexation was based on constructing the CD with the seven identical glucose units positioned symmetrically around the Z-axis, such that all the glycosidic oxygens are in the XY plane and their center was defined as the center of the coordination system [35]. The PM molecule was docked into the cavity of the CD with the central nitrogen atom connecting the two benzene rings that coincides with the Z-axis. Docking was initially done to maximise the electrostatic and hydrophobic interactions between the host and the guest molecules. Multiple starting points were generated by moving the guest molecules along the – and + Z-axis from 10 to −10 Å, at 1 Å intervals, and by rotating the guest molecules from 0°–360° at 45° intervals. Three different inclusion orientations, *i.e.*, the nitrogen from the alkyl group of the PM vertically, facing up and down horizontally into the cavity of the host, were considered for each case (Figure 3).

The inclusion interactions were simulated in vacuum and the presence of water molecules were ignored to save computational time especially for large molecules. The complexation energy, ΔE_comp_, was calculated for the minimum energy structures by the following equation:

(2) $$\Delta E_{\text{comp}}=E_{\text{comp}}-E_{G}-E_{H}$$

where E_comp_, E_G_, and E_H_ represent the total energy of the host-guest complex, the free guest molecule and the free host molecule, respectively. The magnitude of the energy change is an indication of the driving force towards complexation. The more negative the complexation energy change, the more thermodynamically favourable is the inclusion complex.

## 3. Results and Discussion

The response of ionophore-based PS is very much governed by the molecular recognition ability between the analyte (guest) and the host molecule. The interactions between the guests and CDs are mainly facilitated by the hydrophobic interactions of the hydrophobic benzene ring of the guest which is presumably included in the hydrophobic cavity of the CD receptor [36] The formation of inclusion complexes of crown ethers with not only metal ions, but also organic molecules such as amines is also well-known. CDs are cyclic oligosaccharides that consist of d-glucopyranose residues linked to one another with α-1,4-glycosidic bonds. The most frequently used CDs are those containing six, seven and eight α- d-glucose units (commonly referred to as α-, β- and γ-CDs, respectively) with truncated cylindrical molecular shapes providing a hydrophobic cavity [37]. An important property of CDs is their ability to form inclusion complexes with a large number of organic and inorganic compounds—a property that has been exploited in pharmaceutical formulations of certain drugs [36]. Their unique ability to recognise different forms of enantiomers readily lend themselves in the separation of chiral molecules (e.g., in CE) and in chiral drug sensing [23,36].

The mechanism of the membrane response of the solid-state sensor is believed to be as proposed by Bakker and Pretsch [38]. Promethazine cation is transported through the sensing membrane and then to the redox-active monolayer that produces electrons which are measured by the metal platinum electrode.

### 3.1. Effect of Membrane Composition

Initially, the effect of the type of ionophore on the sensor performance was investigated using BEHA as plasticiser. Membranes using α-CD and DB18C6 (sensors 2 and 5, Table 1) resulted in non-functional sensors.

The cavities of these hosts are probably too small [α-CD (174 Å)] [35] to accommodate the guest molecule. When hosts with larger cavity sizes ([β-CD (262 Å), γ-CD (427 Å) and DB30C10] were used, promising PM sensors were obtained. The characteristics of these sensors were further improved by adding KTPB as membrane additive (sensors 10–12), and near Nernstian responses were obtained. Several different membrane compositions that contained 1.0, 1.2, 1.6, and 2.0% of KTPB were also investigated. The best composition of KTPB, resulting in better slope, correlation coefficient and wide concentration range was 1.2%, therefore this amount of KTPB was used in all membranes. The beneficial effects of adding additives such as KTPB have been noted for reducing the membrane resistance and suppressing the permeation of counter anions in aqueous phase into the membrane phase [25,39]. On the contrary, sensors without the additive (sensors 3, 4 and 6) exhibited shorter linear range and produced sub-Nernstian slopes. It has been reported that in the absence of an additional lipophilic anion exchanger, poor performance may be caused by possible trace anionic impurities within the PVC used [39]. Non-functional sensors were obtained with membranes that only contained plasticisers (sensors 7–9). The observed effects of the membrane compositions on the sensor response are clear indicators that inclusion complexes were formed between the larger cavity hosts and the PM guest molecule.

### 3.2. Optimisation of FIA Parameters

#### 3.2.1. Effect of Carrier Stream pH and Concentration

**Figure 4.**
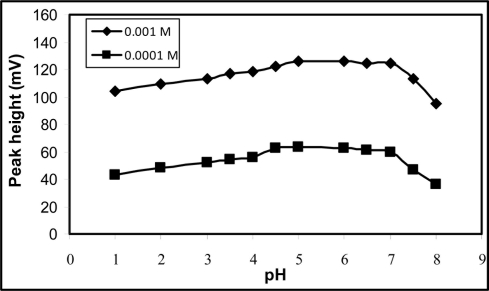
pH profile of sensor no. 10. Carrier stream, 0.1 M acetate buffer; injection loop, 150 μL; flow rate; 3.25 mL min^−1^.

This was studied by changing the carrier stream pH from 1.0 to 8.0 and measuring the peak heights of injected standard solution of PM. The peak height increases steadily over pH 1.0 to 4.5 but remains constant from pH 4.5 to 7.0, and decreases abruptly from pH 7.0 onwards (Figure 4). At pH values below 4.5 the interference of hydronium ion is expected, while at pH above 7.0, reduction in signal is most probably attributed to the formation of the free PM base [18]. The effect of acetate buffer concentration (0.1 to 0.5 M) was also investigated. It was found that there was no significant differences in the peak heights over this concentration range. Thus, a carrier stream of 0.1 M acetate buffer (pH 6.0) was used for the rest of the studies.

#### 3.2.2. Effect of Sample Volume

The effect of injected sample volume (50 to 400 μL) was studied by injecting 1 × 10^−3^ M PM into the FIA unit. Peak heights were found to increase with increasing injected volume but a 150 μL sample loop was used as it provided a good compromise between sensitivity and speed of analysis.

#### 3.2.3. Effect of Flow Rate

1 × 10^−3^ M PM was injected under different flow rates (0.74 to 4.55 mL min^−1^) using a carrier stream of 0.1 M acetate buffer (pH 6.0) and 150 μL injection loop. It was found that, as the flow rate increased, the peaks became higher and narrower until a flow rate of 2.83 mL min^−1^ was reached. This flow rate was used throughout this work, as higher flow rates resulted in the consumption of higher amounts of solvents and chemicals.

### 3.3. Lifetime

**Figure 5.**
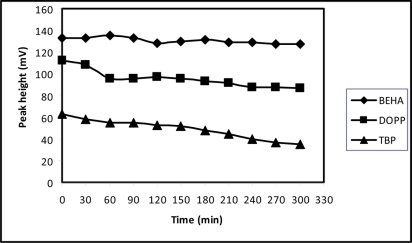
Lifetime studies of promethazine sensors based on β-CD with different plasticizer (sensors 10, 13 and 16). Peak heights from the injection of promethazine standards at regular interval were noted when continuously pumped with carrier stream.

The lifetime of the sensors were studied by continuously pumping for 300 min and repeatedly injecting 1 × 10^−3^ M PM at 30 minute intervals (Figure 5). No noticeable reduction in peak heights over this period was observed for sensor 10. In contrast, sensors that were plasticised with DOPP and TBP (sensors 13 and 16, respectively) experienced a marked reduction in peak height after 60 minutes of continuous use. Indeed, it was found that sensor 10 remained operational over one month of daily use.

### 3.4. Selectivity

Selectivity is an important characteristic of a sensor that delineates the extent to which the device may be used in the estimation of an analyte ion in the presence of other ions and extent of utility of the sensor in real sample measurements. The selectivity coefficient $\left(K_{A,B}^{\text{pot}}\right)$ of the sensor was determined using the separate solution method [27] at a fixed concentration of 1 × 10^−3^ M for both drug and interfering ions. Table 2 shows that there are no significant interferences from the cations, trifluoperazine and the sugars tested. Insignificant interferences from the alkali and alkaline earth metal ions suggest the good prospect of the sensor to be used in biological fluids. The overall selectivity of sensor 10 is better than that of sensors that are based on γ-CD or DB30C10 (sensors 11 and 12).

Under the optimum conditions, response time of not more than 5 s was obtained for an injected sample, translating into a sample throughput of about 96 samples h^−1^. The FIA system was characterised by a dispersion coefficient of 1.9, which was calculated using the equation:

(3) $$D=h_{o}/h$$

where *h*_o_ and *h* is the peak height for undispersed and dispersed sample, respectively. The limited dispersion obtained indicate the limited mixing of analyte and solutions in the flow system, ensuring a sensitive detector output.

### 3.5. Analysis of Real Samples

#### 3.5.1. Determination of PM in Pharmaceutical Formulations

The analytical usefullness of the sensor was tested by determining the concentration of PM in solutions of syrups and tablets. Good agreement of results from the manufacturer’s claimed values, the reference USP and from the proposed FIA sensor was obtained for all the samples tested (Table 3).

#### 3.5.2. Determination of PM in Spiked Human Urine

The capability of the sensor was further tested for the determination of PM in undiluted human urine that had been spiked with three levels of PM. Results obtained are listed in Table 3. The good recoveries demonstrate the applicability of the sensor for routine analysis without prior sample pretreatment.

### 3.6. Molecular Modelling

**Figure 6.**
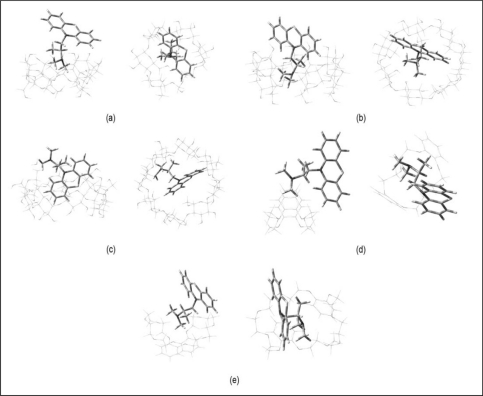
The energy minimised structures obtained from PM3 calculations for the side and top view of **(a)** promethazine/α-CD, **(b)** promethazine/β-CD, **(c)** promethazine/γ-CD, **(d)** promethazine/DB18C6, and **(e)** promethazine/DB30C10 complexes.

To further understand how molecular recognition takes place at the atomic level, molecular modelling techniques were used to complement the experimental studies. The complexation energies (E_comp_) of the PM inclusion complexes are listed in Table 4. The optimised geometries for the lowest energy conformation for the inclusion complexes of PM with CDs and dibenzo-crown ethers are shown in Figure 6, respectively. In general, the inclusion complexes of CDs with PM have larger binding energies when compared to their dibenzocrown ethers counterpart. The overall lowest binding energies with the different hosts decrease in the order of: γ-CD > β-CD > α-CD > DB30C10 > DB18C6.

The dominant driving force for the complexation is evidently H-bondings interaction. Close observation on the structure of the inclusion complexes shows that γ-CD complexes have more H-bonding interactions compared to the β-CD complexes. Various authors have also reported that intermolecular hydrogen bondings between the host-guest interactions resulting from the fitting of small molecules into the CD cavities play an important role in the binding energies of a number of CD inclusion complexes [40,41]. An almost complete inclusion of the guest molecule was observed for the promethazine/γ-CD complex (Figure 6(c)). Meanwhile, for the promethazine/β-CD complex, only the N-alkyl group is included leaving the aromatic rings protrude outside the cavity (Figure 6(b)). However, for the α-CD the N-alkyl group barely touches the upper rim of the α-CD (Figure 6(a)). Consistently, no H-bondings were observed for α-CD and that of the crown ether complexes. The high and positive complexation energy displayed by promethazine/DB18C6 complex (Table 4), shows that inclusion complex between the two molecules are highly unfavourable from the theoretical point of view, consistent with the experimental observations.

## 4. Conclusions

Good quality PM potentiometric sensors were realised using some relatively large cavity size ionophores such as β-CD, γ-CD and DB30C10. The sensor based on β-CD, plasticised with BEHA and containing KTPB additive offers the best overall sensor characteristics, especially in terms of selectivity. The integration of the sensor to a FIA system resulted in a selective device that enables the rapid determination (sample throughput, 96 samples h^−1^) of PM in pharmaceutical preparations and human urine. The proposed FIA system is superior to the other analytical techniques based on HPLC [3,4] as it is not only rapid but uses very simple, and cheap instrumentation. Other advantageous features of potentiometric-type sensors such as the ability to function even in turbid and coloured environments have been well-noted. Although the molecular modelling work that was carried out was somewhat simplistic in the sense that interactions from the plasticiser was excluded, nevertheless they provide valuable insights into the nature of interactions between the hosts and guest molecules.

## Figures and Tables

**Table 1.** Effect of membrane composition on the characteristics of promethazine sensors.

**Sensor No.**	**Plasticiser, %**	**Ionophore**	**Additive**	**Slope (mV decade^−1^)**	**Correlation coefficient**	**Linear range (M)**	**LOD (M)**
1	BEHA (65.8)	-	KTPB	48.6	0.9950	5 × 10^−5^−1 × 10^−2^	5 × 10^−5^
2	BEHA (66.0)	α-CD	-	1.12	-	-	-
3	BEHA (66.0)	β-CD	-	50.9	0.9938	1 × 10^−4^−1 × 10^−2^	9 × 10^−5^
4	BEHA (66.0)	γ-CD	-	52.1	0.9972	1 × 10^−4^−1 × 10^−2^	7 × 10^−5^
5	BEHA (66.0)	DB18C6	-	Unstable	-	-	-
6	BEHA (66.0)	DB30C10	-	49.1	0.9991	1 × 10^−4^−1 × 10^−2^	8 × 10^−5^
7	BEHA (68.0)	-	-	Unstable	-	-	-
8	DOPP (68.0)	-	-	Unstable	-	-	-
9	TBP (68.0)	-	-	Unstable	-	-	-
10	BEHA (64.8)	β-CD	KTPB	61.3	0.9996	1 × 10^−5^−1 × 10^−2^	5 × 10^−6^
11	BEHA (64.8)	γ-CD	KTPB	55.2	0.9976	1 × 10^−5^−1 × 10^−2^	9 × 10^−6^
12	BEHA (64.8)	DB30C10	KTPB	55.8	0.9988	1 × 10^−5^−1 × 10^−2^	1 × 10^−5^
13	DOPP (64.8)	β-CD	KTPB	57.8	0.9973	1 × 10^−5^−1 × 10^−2^	9 × 10^−6^
14	DOPP (64.8)	γ-CD	KTPB	51.1	0.9942	1 × 10^−5^−1 × 10^−2^	9 × 10^−6^
15	DOPP (64.8)	DB30C10	KTPB	47.0	0.9989	5 × 10^−5^−1 × 10^−2^	5 × 10^−5^
16	TBP (64.8)	β-CD	KTPB	52.1	0.9990	1 × 10^−4^−1 × 10^−2^	5 × 10^−5^
17	TBP (64.8)	γ-CD	KTPB	49.5	0.9947	1 × 10^−4^−1 × 10^−2^	5 × 10^−5^
18	TBP (64.8)	DB30C10	KTPB	40.2	0.9946	1 × 10^−4^−1 × 10^−2^	7 × 10^−5^

**Table 2.** Selectivity coefficients of promethazine sensors towards cations, trifluoperazine and sugars.

**Foreign ion**	**log**$K_{A,B}^{\text{pot}}$		
	**Sensor 10**	**Sensor 11**	**Sensor 12**
Li^+^	−3.87	−3.66	−3.89
Na^+^	−4.06	−3.77	−2.42
K^+^	−3.58	−2.93	−2.06
Mg^2+^	−4.42	−3.71	−3.08
Ca^2+^	−3.83	−4.25	−3.28
Co^2+^	−4.77	−3.33	−2.95
Cu^2+^	−4.29	−4.12	−3.81
Cr^3+^	−4.64	−5.11	−4.86
Fe^3+^	−5.35	−4.98	−5.02
Glucose	−4.61	−4.20	−3.64
Fructose	−5.01	−4.63	−4.12
Trifluoperazine	−2.85	−3.04	−2.20

**Table 3.** FIA determination of promethazine in syrups, tablets and spiked urine samples using sensor no.10.

**Type of Sample (Manufacturer)**	**Taken/M**	**FIA Method**		**USP Method**	
		**% Rec.**	**% RSD^d^**	**% Rec.**	**% RSD^d^**
Promethazine /syrup^a^	1 × 10^−3^	104	2.3	102.9	0.82
(DHA, Singapore)	1 × 10^−4^	102	0.92	99	1.13
Phenergan/oral solution^a^	5 × 10^−4^	100	0.13	99.2	0.54
(Sanofi-Winthrop Industrie, France)	5 × 10^−5^	99.2	0.51	98.4	0.7
Upha Promethazine/syrup^b^	1 × 10^−4^	107	0.84	103.6	0.65
(CCM Pharmaceutical, Malaysia)	5 × 10^−5^	101	1.9	99.0	0.85
Upha Promethazine/tablets^c^	5 × 10^−3^	101	0.77	98.0	0.6
(CCM Pharmaceutical, Malaysia)	1 × 10^−4^	99.4	0.53	98.4	0.48
Urine 1	1 × 10^−3^	99	1.04	-	-
Urine 2	1 × 10^−4^	97.5	1.49	-	-
Urine 3	1 × 10^−5^	99.2	1.37	-	-

a. PM 5 mg/5 mL.

b. PM 15 mg/5 mL.

c. PM 25 mg/tablet.

d. Average of six determinations.

RSD, relative standard deviation

Rec., Recovery

**Table 4.** Molecular modelling results of interaction energies (*E*_comp_ in kcal mol^−1^) of the optimal configurations of (1:1) promethazine/CDs and promethazine/dibenzocrown ether inclusion complexes.

**Host**	**E_comp_ (kcal mol^−1^)**
α-CD	−17.134
β-CD	−20.384
γ-CD	−21.378
DB18C6	81.467
DB30C10	−16.826
